# Hepato-Nephrocitic System: A Novel Model of Biomarkers for Analysis of the Ecology of Stress in Environmental Biomonitoring

**DOI:** 10.1371/journal.pone.0132349

**Published:** 2015-07-21

**Authors:** Fábio Camargo Abdalla, Caio Eduardo da Costa Domingues

**Affiliations:** Department of Biology, Laboratory of Structural and Functional Biology, Federal University of São Carlos, Sorocaba, São Paulo State, Brazil; Federal University of Rio de Janeiro, BRAZIL

## Abstract

*Bombus* presents a serious global decline of populations and even loss of species. This phenomenon is complex and multifactorial: environmental degradation due to increasing cultivation and grazing areas, indiscriminate use of agrochemicals, and a plethora of xenobiotics daily discharged in the environment. We proposed that bees have an integrated cell system, which ensures protection against chemical stressors up to a certain limit. Therefore, this hypothesis was tested, exposing workers of *Bombus morio* to cadmium, a harmful trace metal nowadays widespread in our society. The workers were kept in BOD (26°C, RH 70%, in the dark), fed *ad libitum*, and divided into a control group (n = 20) and an experimental group (n = 20). For the first group, we offered 2 mL of distilled water; for the experimental groups, 2mL of cadmium at 1 ppb. In relation to the control group, exposed bees showed that their fat body and hemocytes responded in synchronization with pericardial cells in a topographical and temporal cascade of events, where the fat body is the first barrier against xenobiotics, followed by pericardial cells. The immune cells participate throughout the process. To this system, we proposed the name of hepato-nephrocitic system (HNS), which may explain many phenomena that remain unclear in similar research with *Apis mellifera* and other species of bees, as shown in this paper. The bee’s HNS is a system of highly responsive cells to toxicants, considered a novel parameter for the study of the ecology of stress applied in environmental management.

## Introduction

The fat body of insects consists of two main types of cells: trophocyte and oenocyte. Although these cells are apparently associated in a mass of tissue, the former has mesodermal origin, and the latter, ectodermal, presenting different morphology and function [1–3]. Additionally, the insect fat body presents other cell specializations, such as urocytes, hemoglobin cells, mycetocytes, and chromatocytes [4,5]. The photocytes of some Lampyridae species might also be derived from the fat body cells during evolution [6,7]. The fat body is divided into a perivisceral portion, which surrounds the viscera and occupies the insect abdominal cavity, and a parietal portion, which is located adjacent to the insect integument [1–3]. However, there is a specific region where the fat body is associated with two or more different cellular types, playing the role of only one system. Such association occurs along the insect heart in the dorsal vessel, and in bees it may play functions homologous with the hepatocytes and nephrocytes of vertebrates [8].

The fat body and pericardial cells surround all the abdominal portion of the dorsal vessel, but are more abundant in the area of the ostioles, which are openings into the heart valve or *sinus pericardium* and among which the immune system cells are spread. Ostioles originate from symmetrical invagination of parallel muscle walls, which also originates the heart valve. The function of pericardial cells in bees is unknown for sure. However, in *Drosophila melanogaster* they supposedly have a role in filtering the hemolymph, thus controlling the quantity of substances present in the hemocele liquid. Pericardial cells present pinocytic and phagocytic activities and can also have excretory function [9]. The pericardial cells, the urocytes of the fat body, and the Malpighian tubule form the nephrocitic system of bees. Additionally, pericardial cells may be related to the regulation of heart contraction. According to Fife *et al*. (1987), they produce polypeptides found in the hemolymph. The first researchers to suggest a hypothesis for the function of this cell were Mills and King (1965), who proposed that pericardial cells are involved in the metabolism and excretion of hemocele molecules [9,10]. According to Das *et al*. (2008), the function of these cells is still unclear. However, the authors noticed that after removing them and inducing pericardial cell death in *D*. *melanogaster* larvae the specimens presented lower lifetime and more susceptibility to toxins [11]. Currently, pericardial cells are believed to participate in the absorption of toxicants. These substances neutralized by pericardial cells are released back into the hemolymph and can be filtered by the Malpighian tubule [9–13].

Sudden decline and loss of entire populations of several species of the *Bombus* are recorded annually, especially in the European and American continents [14–16]. In Brazil, specifically in the state of Paraná and in some locations in the state of Santa Catarina, Martins & Melo (2010) reported the decline, followed by disappearance, of the species *B*. *bellicosus*. Although the causes for such drastic decrease in the species’ population are diverse, all scientists agree that global warming, intensive agricultural activity, and industrialization play a major role in this phenomenon, drastically exposing the bees to xenobiotics and disturbing the natural balance between bees and their predators [14–19].

Regarding the above, we investigated the effect of the harmful trace metal cadmium. The trace metal is detected in the air, soil and water, and also in organic and inorganic crops, as well as in fruits, vegetables, and cereals in organic crops [20]. For such purpose, we analyzed specific internal organs of the bumble bee *Bombus morio*. These bees are important bioindicators of the environmental condition, thus the relevance of studying their internal organs as sensitive biomarkers of environmental stress.

## Material and Methods

*Bombus morio* workers (Swederus, 1787) were collected in remaining fragments of semi-deciduous forest and Cerrado (23°34’53.1”S 47°31’29.5”) of the Federal University of São Carlos, at the Sorocaba Campus, in the municipality of Sorocaba, state of São Paulo, Brazil. For the areas utilized there was not necessary a formal permission form or ethical form filling. Tree samplings were carried out from summer through early winter (March to July, 2014), 18 specimens of *B*. *morio* were collected on flowers of *Cassia* sp. aff. mannii Aubrev., in the morning (8–11 am) and other two bees were collected on flowers of *Macroptilium atropurpureum* (DC.) Urb. in the afternoon (5–6 pm), using entomology nets. Immediately the collection of one bee, they were identified and placed to Falcon flasks with small perforation on the lid and then, the flasks were transferred for a back box. The workers were kept individually in plastic box with two feeders in BOD (26°C, RH 70%, in the dark), fed *ad libitum* with a mixture of honey, dehydrated pollen and organic soy flour, and divided into control group (n = 20) and experimental group (n = 20). For the first group we offered 2 mL of distilled water; for the experimental groups, 2mL of cadmium 1 ppb. After bee exposure (two days both groups), they were sacrificed after cooling them at 4°C for 30 min. Their dorsal vessel was dissected, fixed in 4% paraformaldehyde in 0.2 M sodium phosphate buffer, pH 7.4. After fixation, the material was embedded in resin JB—4 (Polysciences) according to manufacturer’s recommendations. The resin was polymerized at room temperature [21]. Histological sections of 2 μm thickness were cut with a Leica microtome (RM 2255). The material was stained with hematoxylin and eosin, and analyzed with a Leica photomicroscope (DM 1000). The area of 700 pericardial cells were measured in five individuals using the software LAS (Leica). The sample was analyzed by GraphPad Prism 6.0 statistic software to verify the total variance of the measurements between control and experimental groups.

## Results and Discussion

### Relationship among Dorsal Vessel, Fat Body, Pericardial Cells and Hemocytes in Bees

**Fig 1:**
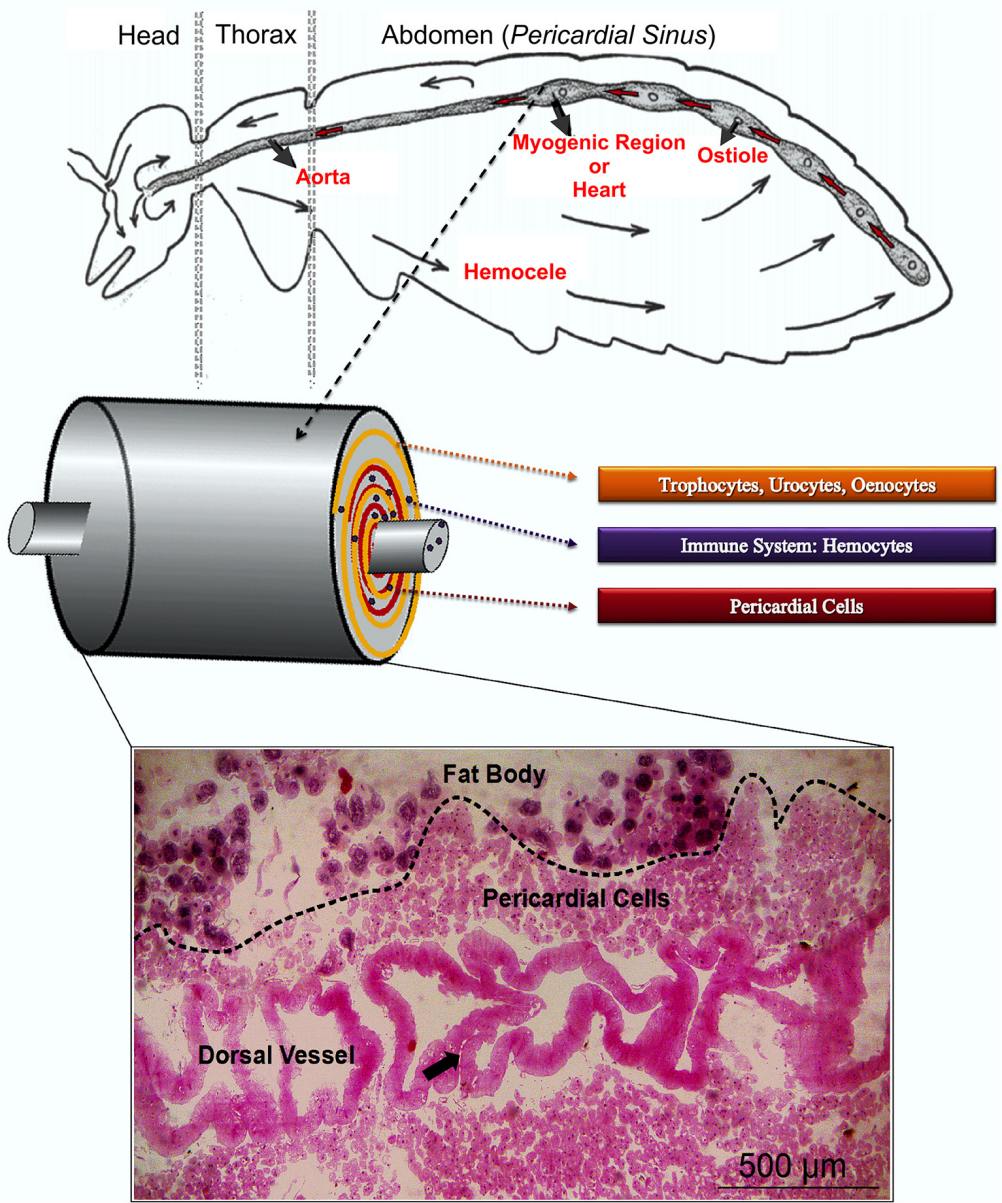
Scheme and micrograph of the hepato-nephrocitic system. In the micrograph the arrow shows the heart valve.

The fat body form an outer layer adjacent to an inner layer of pericardial cells, which covers the entire dorsal vessel. [13]. In regions where there are openings in the dorsal vessel, i.e., ostioles, and in the myogenic region of the dorsal vessel, or heart, the perivisceral fat body composes an organized system with the pericardial cells and hemocytes, i.e., the cells of the immune system. This combination of tissues has a specific topography consisting of an outer layer of fat body involving an inner layer of pericardial cells, forming a concentric system around the entire dorsal vessel. The hemocytes are found within the spaces between the cells of both tissues, or close to the outer wall of the dorsal vessel, or even within it. Therefore, before entering the dorsal vessel, the hemocele must pass through these three systems in a sequential fashion: first through the fat body cells, then through the pericardial cells, finally to enter the dorsal vessel (Fig 1). The hemocele is in contact with the hemocytes all along this system [8].

Despite the name "fat body", in bees this tissue has no relationship with the adipose tissue of vertebrates. The cells of the insect fat body are functionally and structurally distinct from adipocytes [2,5], rather resembling more the hepatic tissue in adults than the adipose tissue. The fat body in insects is related to their main intermediary metabolism [5]. During the larval phase, it is involved in the metabolism of lipids and carbohydrates and in the synthesis and secretion of proteins, which are released in large quantities into the hemolymph in the last larval stage to drive and carry out the metamorphosis [22–28]. Both in pre-embryonic and adult phases, the fat body is involved in the accumulation of toxic materials, such as urate [29–32]. In adult females, it participates directly in vitellogenesis, supplying the soluble precursor for the yolk, i.e., vitellogenin that will be uptaken by the nurse cells of the ovarian follicles in the vitellarium [33–35]. The oenocytes can act in detoxification processes due to the expression of cytochrome P450 NADPH reductase; such enzymes can establish an association with the biotransformation of xenobiotics through oxidative reactions [36–38].

**Fig 2:**
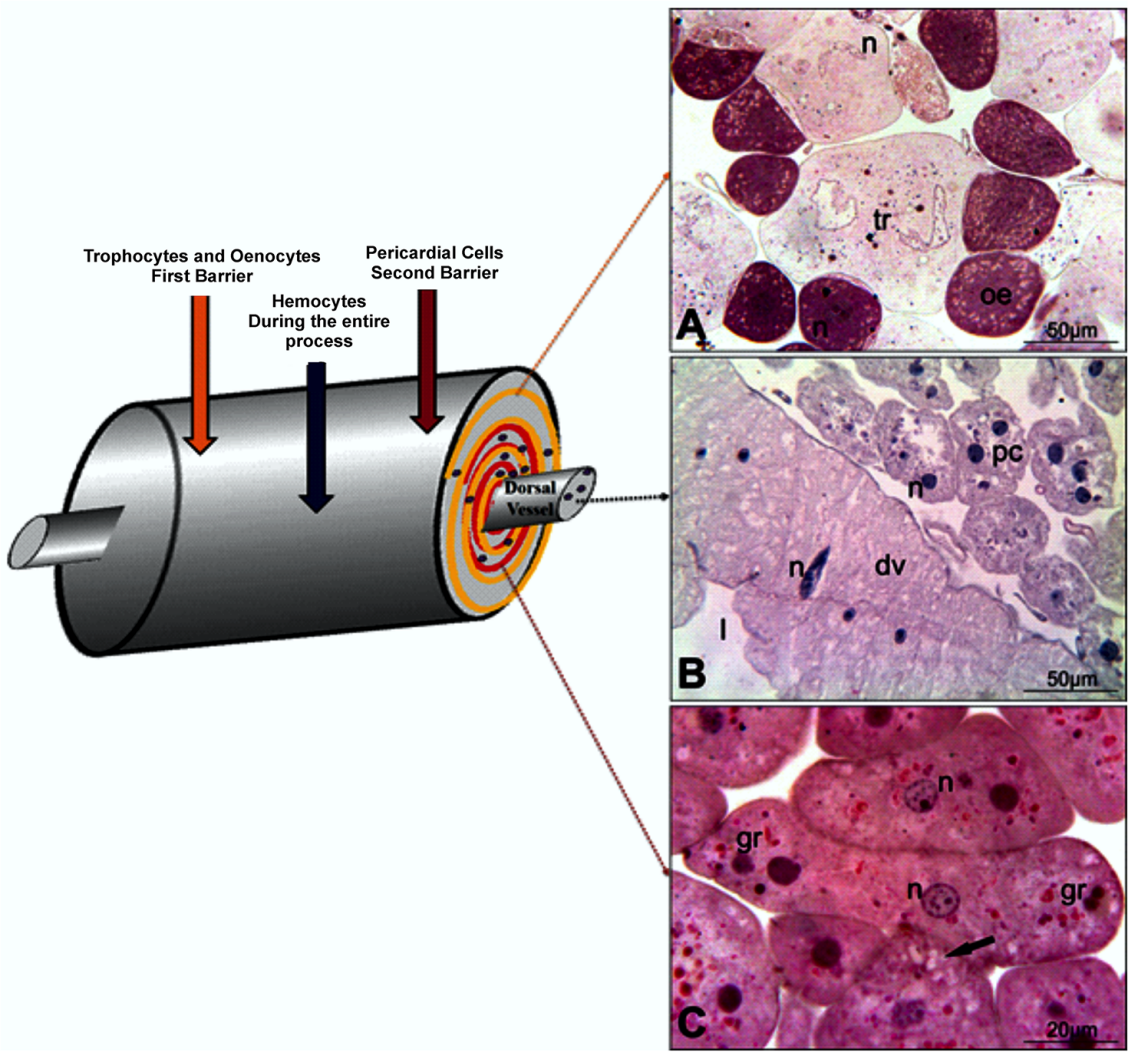
Scheme and micrograph of the hepato-nephrocitic system of the control group. (A) Noticed intact trophocyte (tr) and oenocyte (oe) in close association. (B) Pericardial cells (pc) forming a layer around the dorsal vessel (dv). (C) Detail of the cordonal pericardial cell in stage I, showing intracytoplasmatic vacuoles (arrow) and some eosinophil granules (gr). n = nucleus, l = lumen.

When bees are exposed to xenobiotics, in this case to cadmium, they show that their fat body and immune cells respond in synchronization with the pericardial cells in a topographical and temporal cascade of events, where the fat body is the first barrier against xenobiotics, followed by the pericardial cells. The immune cells participate throughout the process. To this system, we proposed the name of hepato-nephrocitic system or HNS (Figs 1 and 2).

### The Action Mechanism of the HNS towards Cadmium Exposure

**Fig 3:**
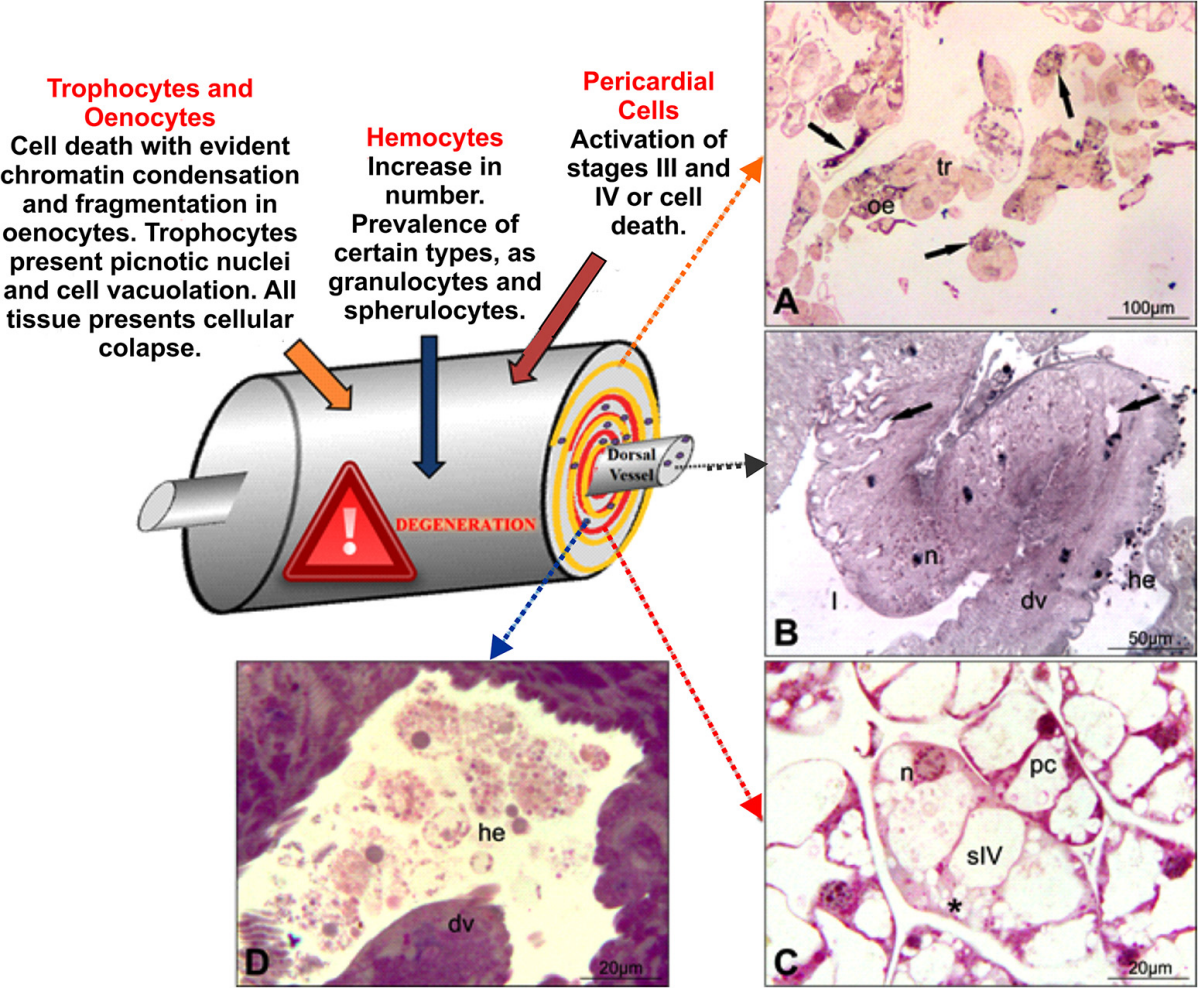
Scheme and micrograph of the hepato-nephrocitic system of the experimental group. (A) Noticed that the trophocyte (tr) and oenocyte (oe) degenerated and present signs of cellular death (arrows). (B) The pericardial cells (pc) forming a layer around the dorsal vessel are in stage IV and the dorsal vessel (dv) present signs of degeneration (arrows). (C) Detail of the cordonal pericardial cell in stage IV (sIV), showing a large intracytoplasmatic vacuole that occupies all the cytoplasm (asterisk) and the nucleus (n) located at the cell periphery. (D) Large quantity of different types of hemocytes (he) in the dorsal vessel (dv). l = lumen.

According to their activity, pericardial cells may show four morphological configurations [9]. The first is characterized by cells having a rounded, central nucleus and peripheral small vacuoles in the cytoplasm (Fig 2C). In stage I, the epithelioid or row arrangement of these cells is very characteristic. In stage II, the vacuoles increase by coalescence of small peripheral vacuoles. Stage III is characterized by a larger central vacuole and few small peripheral ones. In stage IV, the cells are larger, with an irregular contour and a large vacuole occupying almost the entire cell, which causes the displacement of the nucleus towards the periphery of the cytoplasm. In this stage, the uptake of substances from the hemolymph is fully active in these cells (Fig 3C).

According to our results and in relation to the control group, in a brief exposure period (2 days of exposure) the cadmium provoked an intense activity of the pericardial cells, almost all the cells were in stage IV or even in autophagy process [39]; such a phenomenon is evident in observing the cell morphology (Fig 3A). The hemocytes in the control group are scarce both inside and outside the dorsal vessel (Fig 2B), whereas in the bees exposed to cadmium their presence and diversity increased considerably (Fig 3D).

**Fig 4:**
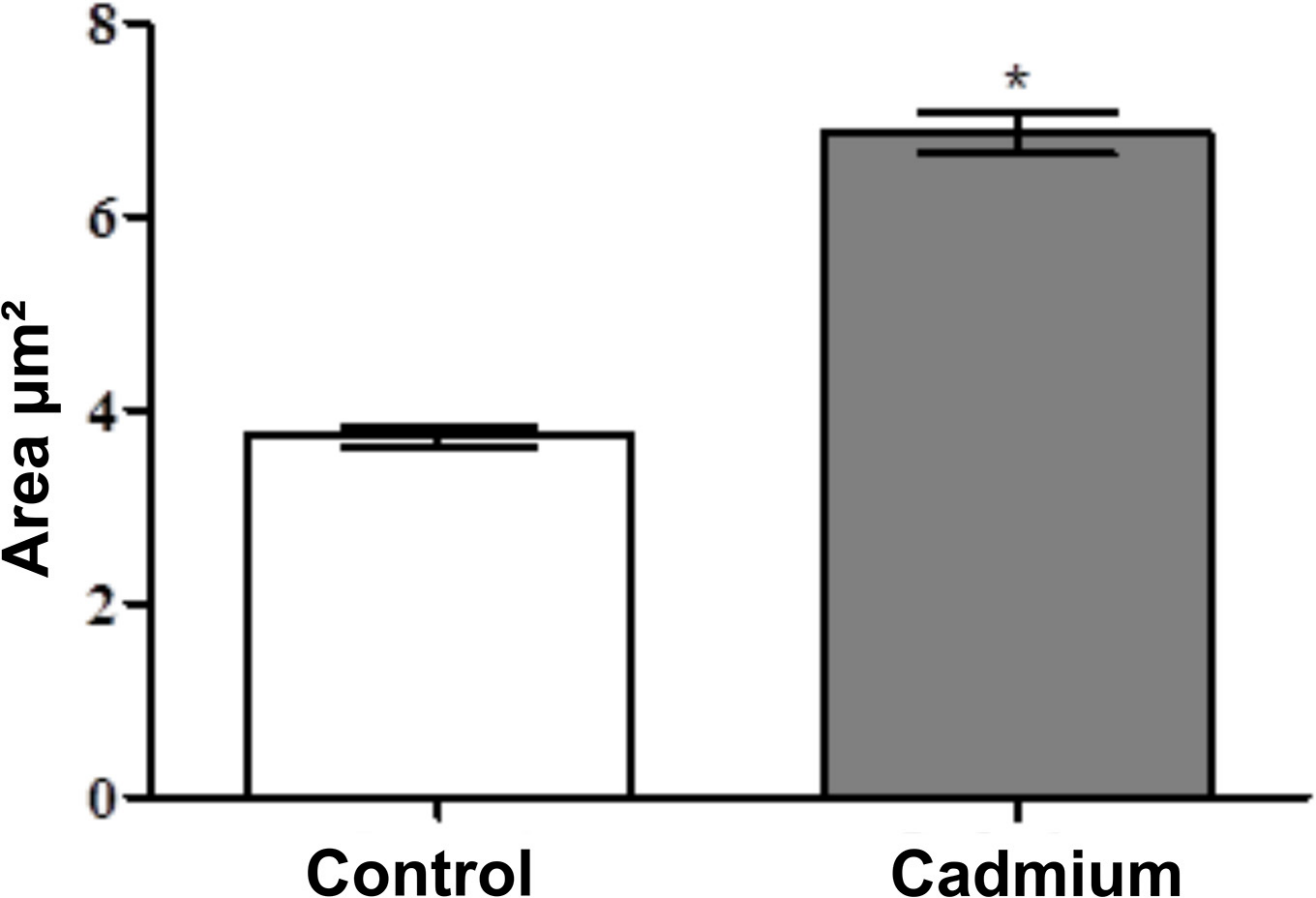
Histogram graph showing the area’s variance of the pericardial cells in relation to the control group. *Statistically different at P > 0.01, Kruskal-Wallis Test.

From our observations with other species and xenobiotics (unpublished data), the first target of xenobiotics in the HNS is the fat body tissue, especially trophocytes and oenocytes. Oenocytes are much more sensible to xenobiotics than trophocytes, presenting incontestable signs of death by apoptosis (Figs 2A and 3A), whereas trophocytes show first cell degeneration then a kind of physiologic cellular death [13]. Control bees present intact fat body, even after 60 days in BOD. Curiously, we cannot count the number of hemocytes in the hemolymph of the experimental groups because the hemolymph seems to be dry in comparison with the control group. Such a phenomenon may be related to hemolymph absorption by pericardial cells, which almost doubled in size in the experimental group as compared to the control group (Fig 4).

For the above, we observed that if the fat body tissue collapses, the pericardial cells are activated. These events do not occur at the same time. First, the fat body cells collapse and cell death occurs, then the pericardial cells are activated, reaching stages III or IV, depending on the xenobiotic (unpublished data). In the case of cadmium, stage IV and some cells in autophagy. Cadmium, even at 1 ppb, causes a high increase in hemocyte number, i.e., the immune system of bees. The immune system activation seems to be constant throughout the whole intoxication process. If the pericardial cells reach stage IV, after fat body collapse, the muscle walls of the dorsal vessel are disrupted (Fig 3B) and the bees are already dead.

## Conclusion

The hepato-nephrocitic system (HNS) of *Bombus morio* may explain many phenomena that remain unclear in similar researches with other species of bees. The bee’s HNS is a system of highly responsive cells to toxicants, considered a novel biomarker parameter for the study of the ecology of stress applied in environmental management. The cadmium concentration used in this work is considered environmentally safe in class 1 and 2 waters by the Brazilian Environmental Council.
